# Involvement of Endoplasmic Reticulum Stress, Autophagy, and Apoptosis in Advanced Glycation End Products-Induced Glomerular Mesangial Cell Injury

**DOI:** 10.1038/srep34167

**Published:** 2016-09-26

**Authors:** Chih-Kang Chiang, Ching-Chia Wang, Tien-Fong Lu, Kuo-How Huang, Meei-Ling Sheu, Shing-Hwa Liu, Kuan-Yu Hung

**Affiliations:** 1 Institute of Toxicology, College of Medicine, National Taiwan University, Taipei, Taiwan; 2 Department of Integrated Diagnostics & Therapeutics, College of Medicine and Hospital, National Taiwan University, Taipei, Taiwan; 3 Department of Pediatrics, National Taiwan University Hospital, Taipei, Taiwan; 4 Department of Urology, College of Medicine, National Taiwan University, Taipei, Taiwan; 5 Institute of Biomedical Sciences, National Chung Hsing University, Taichung, Taiwan; 6 Department of Medical Research, China Medical University Hospital, China Medical University, Taichung, Taiwan; 7 Department of Internal Medicine, College of Medicine, National Taiwan University, Taipei, Taiwan

## Abstract

Advanced glycation end-products (AGEs)-induced mesangial cell death is one of major causes of glomerulus dysfunction in diabetic nephropathy. Both endoplasmic reticulum (ER) stress and autophagy are adaptive responses in cells under environmental stress and participate in the renal diseases. The role of ER stress and autophagy in AGEs-induced mesangial cell death is still unclear. Here, we investigated the effect and mechanism of AGEs on glomerular mesangial cells. AGEs dose-dependently decreased mesangial cell viability and induced cell apoptosis. AGEs also induced ER stress signals in a time- and dose-dependent manner. Inhibition of ER stress with 4-phenylbutyric acid effectively inhibited the activation of eIF2α and CHOP signals and reversed AGEs-induced cell apoptosis. AGEs also activated LC-3 cleavage, increased Atg5 expression, and decreased p62 expression, which indicated the autophagy induction in mesangial cells. Inhibition of autophagy by Atg5 siRNAs transfection aggravated AGEs-induced mesangial cell apoptosis. Moreover, ER stress inhibition by 4-phenylbutyric acid significantly reversed AGEs-induced autophagy, but autophagy inhibition did not influence the AGEs-induced ER stress-related signals activation. These results suggest that AGEs induce mesangial cell apoptosis via an ER stress-triggered signaling pathway. Atg5-dependent autophagy plays a protective role. These findings may offer a new strategy against AGEs toxicity in the kidney.

Diabetes mellitus (DM) is one of the most common metabolic diseases in the world^1^. There are many DM-induced complications such as retinopathy, nephropathy, peripheral neuropathy, and microvascular injury, which accounts for high mortality rates in diabetic patients^1^,^2^,^3^. Advanced glycation end-products (AGEs) resulting from hyperglycemia are reactive derivatives formed by the Maillard reaction or during oxidation of lipids and nucleic acids. AGEs are known to be an important factor in diabetes-induced complications^4^,^5^. AGEs have been found to induce the pancreatic islet endothelial cell apoptosis and skeletal muscle atrophy^2^,^4^. Singh *et al*. also suggested that AGEs play an important role in diabetic nephropathy^6^. In particular, diabetic nephropathy is one of the DM-induced complications, which results in high mortality rates. 25–40% of diabetic patients develop diabetic nephropathy within 20–25 years after the onset of diabetes and over 20% of those diabetic nephropathy patients develop end-stage renal disease in the next 10–20 years^1^,^7^,^8^,^9^. The pathological changes of diabetic nephropathy include glomerular hyperfiltration, mesangial matrix expansion, and glomerular and tubular hypertrophy. There is a high chance that diabetic nephropathy progresses to glomerular sclerosis and renal fibrosis, which cause renal dysfunction^10^,^11^,^12^. Mesangial cells are known to play an important role in diabetic nephropathy progression. Mesangial cells are responsible for modulating glomerular filtration and phagocytosing apoptotic cells and maintaining the structure of glomerular capillary as well as mesangial matrix homeostasis in the kidney^13^. In diabetic nephropathy patients, mesangial matrix expansion, which could cause by AGEs or other diabetic nephropathy injury factors, is one of important mechanism for kidney dysfunction^14^,^15^. Furthermore, mesangial cell apoptosis induced by AGEs or high glucose was another important mechanism in diabetic nephropathy-induced albuminuria and renal dysfunction^13^,^16^,^17^. High glucose can induce oxidative stress and intrinsic proapoptotic pathway in mesangial cells^18^,^19^. AGEs also evoked apoptosis in both mouse and human mesangial cells^20^,^21^. Although the secretions of vascular endothelial growth factor and monocyte chemotactic protein 1 protein have been found to be associated with AGEs-induced mesangial cell apoptosis^20^, the mechanisms of mesangial cell apoptosis induced by AGEs are still unclear.

Endoplasmic reticulum (ER) stress and autophagy have known to be associated with many kinds of renal injuries. Protein synthesis, folding, and modification are major functions of ER in the cells. If ER functions are disturbed, unfolded proteins will accumulate in the cells and subsequently induce ER stress and an adaptive response - unfolded protein response (UPR)^22^,^23^. UPR can protect cells under mild or short-term stress, but induces apoptosis if cells receive server or unrecoverable damage^22^. There are three major signaling pathways in UPR, including PKR-like ER kinase (PERK)-Eukaryotic initiation factor 2α (eIF2α), activating transcription factor 6 (ATF6), and IRE-1. Three pathways can induce the expression of CCAAT/enhancer-binding protein (C/EBP) homologous protein (CHOP), which plays an important role in ER stress-induced apoptosis^24^. Previous study showed that CHOP expression enhanced cell apoptosis in renal ischemia reperfusion injury^25^. Xu Y *et al*. found that ER stress regulated transforming growth factor (TGF)-β-induced mesangial cell injury^26^. However, the role of ER stress in AGEs-induced mesangial cell apoptosis remains to be clarified. On the other hand, autophagy is another adaptive response in the cell under stress condition in which the double-membrane vacuoles (autophagosomes) are formed and delivered the cell contents to lysosome for degradation^27^. Several studies have also demonstrated that autophagy can prevent stress-induced apoptosis in the kidney^28^,^29^,^30^, and ER stress plays an important role in autophagy regulation^31^,^32^. Therefore, we hypothesized that both ER stress and autophagy may be involved in the AGEs-induced mesangial cell apoptosis. In this study, we tried to clarify whether ER stress and autophagy are participated in AGEs-induced mesangial cell apoptosis and death.

## Results

### AGEs induced mesangial apoptosis in a dose dependent manner

**Figure 1:**
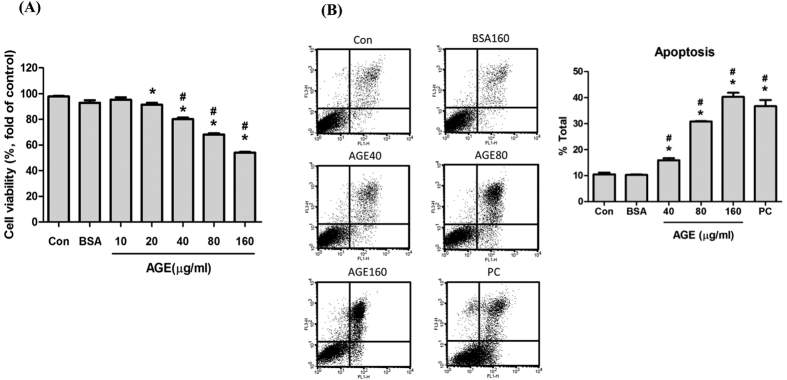
Effects of AGEs on mesangial cell viability and apoptosis induction. Mesangial cells treated with AGEs (10, 20, 40, 80 and 160 μg/ml) for 24 h. BSA (160 μg/ml) was used as a negative control. The cell viability was evaluated by MTT assay (**A**). For apoptosis evaluation, cells were stained with Annexin V and PI after treated with AGEs. Cells were treated with 3% formaldehyde for 30 minutes as a positive control (PC). Percentages of apoptotic cells were determined by flow cytometry (**B**). Data are presented as mean ± SEM of three independent experiments performed in triplicates. *P < 0.05 as compared to control group. ^#^*P* < 0.05 as compared to BSA-treated group.

**Figure 2:**
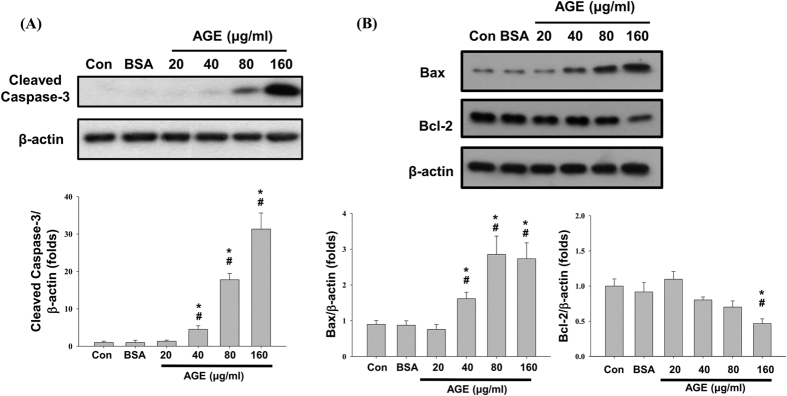
Effects of AGEs on apoptosis-related protein expressions. Cells were treated with AGEs (20–160 μg/ml) for 24 h. BSA (160 μg/ml) was used as a negative control. The cleavage of caspase-3 (**A**) and the protein expressions of Bax and Bcl-2 (**B**) were determined by Western blot. Data are presented as mean ± SEM of three independent experiments performed in duplicates. *P < 0.05 as compared to control group. ^#^*P* < 0.05 as compared to BSA-treated group.

Mesangial cells were treated with various concentrations of AGEs (10, 20, 40, 80, and 160 μg/ml) and BSA (160 μg/ml) for 24 h to evaluate the cell viability. The cell viability significantly reduced in a dose-dependent manner after AGEs treatment as compared with normal control or BSA control (Fig. 1A). To understand whether apoptosis involved in AGEs-reduced cell viability, annexin V/PI staining was used. The results showed that AGEs (40–160 μg/ml) significantly induced apoptosis at in a dose-dependent manner (Fig. 1B). Furthermore, AGEs significantly increased the cleavage of caspase-3 protein (Fig. 2A). AGEs also significantly increased the Bax protein expression at the concentrations of 40–160 μg/ml, but decreased the Bcl-2 protein expression at the concentration of 160 μg/ml (Fig. 2B). These results show that AGEs induce mesangial cell apoptosis in a dose-dependent manner.

### AGEs induced ER stress and autophagy in both dose and time-dependent manners

**Figure 3:**
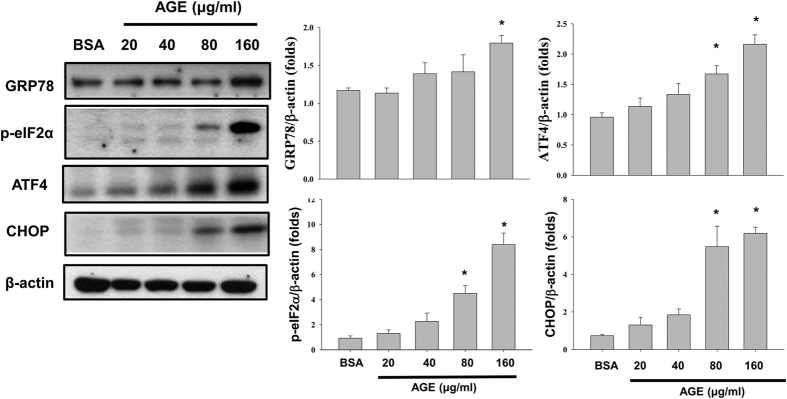
Effects of AGEs on ER stress-related protein expressions. Cells were treated with AGEs (20–160 μg/ml) for 24 h. BSA (160 μg/ml) was used as a negative control. The protein expressions of GRP78, phospho-eIF2α, ATF4, and CHOP were determined by Western blot. Data are presented as mean ± SEM of three independent experiments performed in duplicates. *P < 0.05 as compared to control group. ^#^*P* < 0.05 as compared to BSA-treated group.

**Figure 4:**
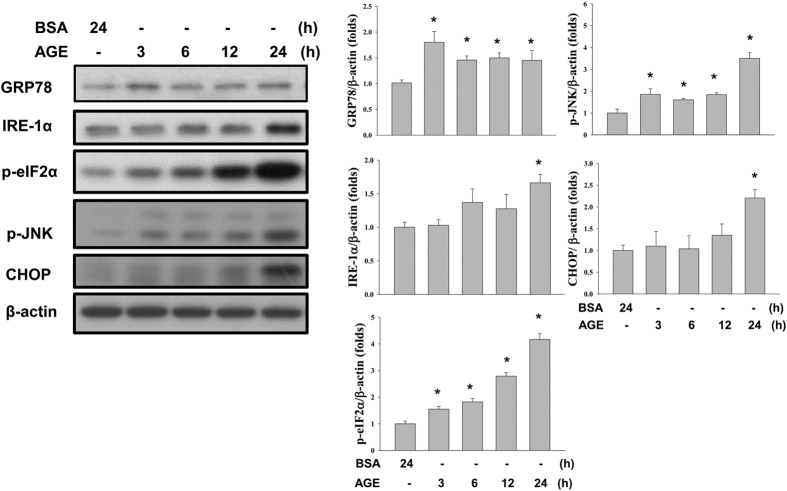
Effects of AGEs on ER stress-related proteins at various time points. Cells were treated with AGEs (160 μg/ml) for 3, 6, 12, and 24 h. BSA (160 μg/ml) was used as a negative control. The protein expressions of GRP78, IRE-1α, phospho-eIF2α, phospho-JNK, and CHOP were determined by Western blot. Data are presented as mean ± SEM of three independent experiments performed in duplicates. *P < 0.05 as compared to control group. ^#^*P* < 0.05 as compared to BSA-treated group.

To understand whether ER stress involved in AGEs-induced mesangial cell apoptosis, ER stress-related proteins were detected by Western blots. Mesangial cells were treated with various concentrations of AGEs (20, 40, 80, and 160 μg/ml) and BSA (160 μg/ml) for 24 h. AGEs significantly upregulated the protein expressions of GRP78, ATF4, and CHOP and phosphorylation of eIF2α in a dose-dependent manner (Fig. 3). AGEs also increased the phosphorylations of eIF2α and c-Jun N-terminal kinase (JNK) and the protein expressions of GRP78, IRE-1α, and CHOP in a time dependent manner (Fig. 4). These results indicate that AGEs induced ER stress in mesangial cells, which may participate in AGEs-induced mesangial cell apoptosis.

**Figure 5:**
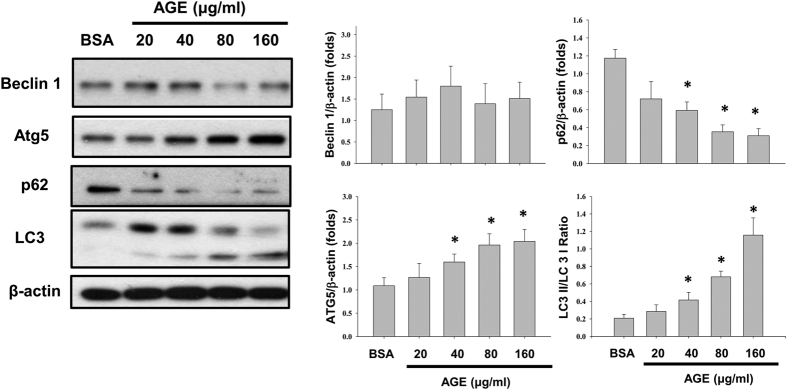
Effects of AGEs on autophagy-related protein expressions. Cells were treated with AGEs (20–160 μg/ml) for 24 h. BSA (160 μg/ml) was used as a negative control. The cleavage of LC3 and the protein expressions of Atg 5, beclin 1, and p62 were determined by Western blot. Data are presented as mean ± SEM of three independent experiments performed in duplicates. *P < 0.05 as compared to BSA-treated group.

**Figure 6:**
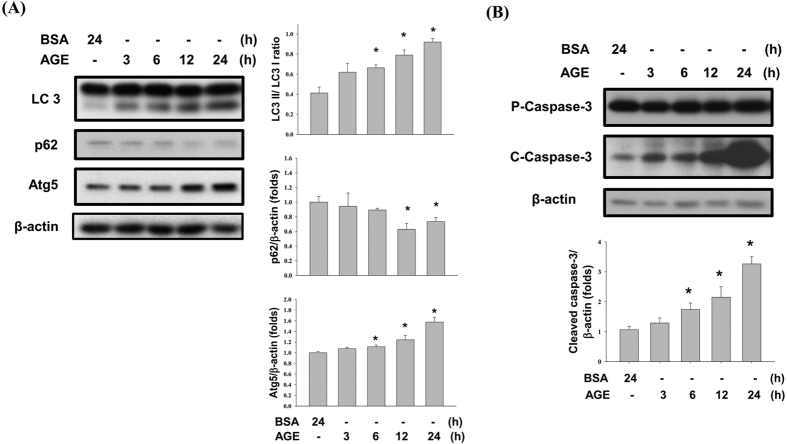
Effects of AGEs on autophagy and apoptosis at various time points. Cells were treated with AGEs (160 μg/ml) for 3, 6, 12, and 24 h. BSA (160 μg/ml) was used as a negative control. The protein expressions of LC3, p62, Atg5, and CHOP (**A**) and caspase-3 (**B**) were determined by Western blot. Data are presented as mean ± SEM of three independent experiments performed in duplicates. *P < 0.05 as compared to control group. ^#^*P* < 0.05 as compared to BSA-treated group.

Autophagy is also an important mechanism involved in different renal injuries. AGEs significantly increased LC3 cleavage (LC3-II/LC3-I ratio) and autophagy-related gene 5 (Atg5) protein expression in mesangial cells in a dose- and time-dependent manner (Figs 5 and 6A). The protein expression of beclin1 was not affected by AGEs treatment (Fig. 5). The p62 protein, which is one of downstream signals of autophagy, was also decreased after AGEs treatment in a dose- and time-dependent manner (Figs 5 and 6A). Furthermore, the time course of caspase-3 activation was similar with the expression of ER stress and autophagy-related proteins after AGEs treatment (Fig. 6B). These results suggest that AGEs accumulation in mesangial cells induces autophagy activity.

### 4-Phenylbutyric acid (4PBA) reversed AGEs-induced ER stress and apoptotic response in mesangial cells

**Figure 7:**
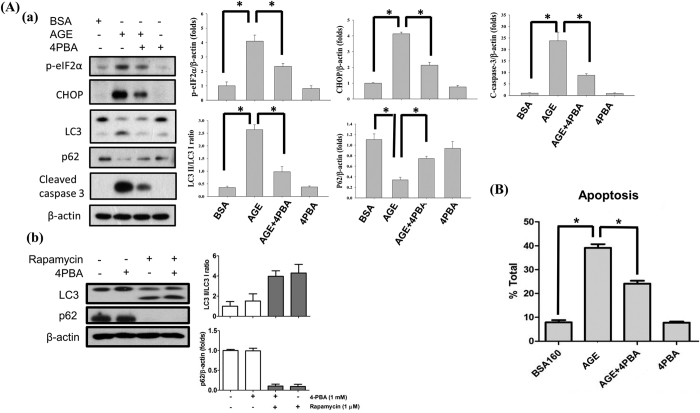
The role of ER stress in AGEs-induced mesangial cell apoptosis. Cells were treated with AGEs (160 μg/ml) with or without ER stress inhibitor 4-phenylbutyric acid (4PBA, 1 mM) treatment for 24 h. The protein levels of phospho-eIF2α, CHOP, LC3, and cleaved caspase-3 were determined by Western blot (A-a). In some experiments, the effect of 4PBA on rapamycin-induced autophagy was tested (A-b). The percentages of apoptotic cells were determined by PI-Annexin V staining (**B**). Data are presented as mean ± SEM of three independent experiments performed in duplicates (**A**) or triplicates (**B**). *P < 0.05 as compared to BSA-treated group or AGEs-treated group.

4PBA is a potent ER stress inhibitor, which can attenuate ER stress-induced cell injury^33^,^34^. We tried to use 4PBA to inhibit AGEs-induced ER stress and figure out the role of ER stress in AGEs-induced mesangial cell apoptosis. Mesangial cells were treated with AGEs (160 μg/ml) with or without 4PBA (1 mM) for 24 h. Co-treatment with 4PBA significantly reversed AGEs-induced CHOP protein expression and eIF2α phosphorylation and caspase-3 cleavage (Fig. 7Aa). Annexin V/PI staining also showed that 4PBA significantly reduced the percentage of AGEs-induced apoptotic mesangial cells (Fig. 7B). Moreover, co-treatment with 4PBA significantly reversed AGEs-induced LC3 cleavage (decrease of LC3-II/LC3-I ratio) and p62 protein degradation (Fig. 7Aa). 4PBA by itself did not affect the rapamycin-induced autophagy in mesangial cells (Fig. 7Ab). These results indicate that AGEs may induce caspase-3 activation and apoptosis and autophagy through an eIF2α/CHOP signaling pathway.

### Inhibition of Atg5 expression aggravated AGEs-induced injury in mesangial cells

**Figure 8:**
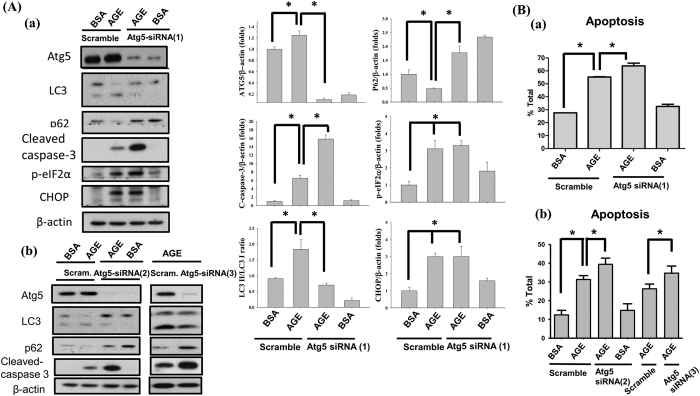
The role of autophagy in AGEs-induced mesangial cell apoptosis. Mesangial cells were transfected with single Atg5 siRNA [(MSS247019; siRNA (1), (A-a)] or mixture of Atg5 siRNAs [(MSS247019, MSS247020, and MSS247021; siRNA (2), (A-b)] or single Atg5 siRNA [(MSS247020; siRNA (3), (B-a)] or scramble control before AGEs treatment. Cells were treated with BSA (160 μg/ml) or AGEs (160 μg/ml) for 24 h. The protein levels of Atg5, p62, LC3, phospho-eIF2α, CHOP, and cleaved caspase-3 were determined by Western blot (**A**). The percentages of apoptotic cells were determined by PI-Annexin V staining (**B**). Data are presented as mean ± SEM of three independent experiments performed in duplicates (**A**) or triplicates (**B**). *P < 0.05 as compared to BSA-treated group or AGEs-treated group.

To clarify the role of autophagy in AGEs-induced mesangial cell apoptosis, cells were transfected with scramble or Atg5 siRNA for 6 h before AGEs treatment. Transfection with Atg5 siRNA (MSS247019) significantly reduced Atg5 protein expression and LC3 cleavage and p62 protein degradation (Fig. 8Aa). To confirm the effect of Atg5 knockdown, a single Atg5 siRNA (MSS247020) and a mixture of three Atg5 siRNAs (MSS247019, MSS247020, and MSS247021) were used. As shown in Fig. 8Ab, Atg5 siRNAs (MSS247020 and mixture) could also reduce Atg5 protein expression and LC3 cleavage and p62 protein degradation. These results indicate that Atg5 siRNA transfection inhibits AGEs-induced autophagy. Moreover, the AGEs-induced cleavage of caspase-3 was significantly enhanced by Atg5 siRNAs transfection (Fig. 8Aa,b). Similarly, transfection of Atg5 siRNAs significantly enhanced AGEs-induced cell apoptosis (Fig. 8Ba,b). These results may not due to the off-target effect of the Atg5 knockdown. However, transfection of Atg5 siRNA did not alter the protein expression of CHOP and the phosprylation of eIF2α in AGEs-treated mesangial cells (Fig. 8Aa). These results suggest that autophagy may play a protective role in AGEs-induced mesangial cell apoptosis. ER stress is capable of interfering with the function of autophagy in mesangial cells.

## Discussion

In this study, we found that AGEs induced renal glomerular mesangial cell apoptosis and death. We further found that both ER stress and autophagy were involved in AGEs-induced mesangial cells apoptosis. Inhibition of ER stress by 4PBA decreased eIF2α phosphorylation, CHOP protein expression, and cell apoptosis in AGEs-treated mesangial cells. Inhibition of autophagy by Atg5 siRNA transfection decreased Atg5 protein expression, LC3 cleavage, and p62 degradation, but aggravated cell apoptosis in AGEs-treated mesangial cells. Furthermore, ER stress inhibition could reduce the autophagy response, but autophagy inhibition did not affect ER stress activation. These results suggest that ER stress is the upstream regulator of autophagy in mesangial cells exposed to AGEs, and autophagy may play a protective role in AGEs-induced mesangial cell apoptosis.

The studies have shown that the serum levels of AGEs are significantly increased in the diabetic patients versus the control subjects: type 1 DM, 6.6 (5.1–9.9) vs. 5.5 (3.7–8.2) U/ml^35^; type 2 DM, 7.4 (4.4–10.9) vs. 4.2 (1.6–6.4) U/ml (1 AGE unit = 1 μg/mL AGE-BSA standard)^36^. The blood AGEs levels were also increased in the diabetic mouse models: control mice, 3–6 μg/ml; streptozotocin-induced diabetic mice, 65–75 μg/ml; high-fat, high-fructose-induced diabetic mice, 10–15 μg/ml^37^; *db/db* diabetic mice, 50–60 μg/ml^38^. AGEs have been reported to decrease cell viability and induce apoptosis in various cell types. Yamagishi *et al*. observed that AGEs (AGE-BSA) at 100 μg/ml reduced the viable cell numbers of retinal pericytes and induced apoptotic cell death in pericytes at 250 μg/ml^39^. Lan *et al*. also found that AGEs (AGE-BSA, 25–200 μg/ml) induced apoptosis in pancreatic islet endothelial cells^2^. Mahali *et al*. have demonstrated that AGEs [AGE-human serum albumin (HSA)] at 100 μg/ml induced apoptosis in some cancer cell lines^40^. Geoffroy *et al*. have shown that AGEs (AGE-BSA) at concentrations of <1 μM increase the rat mesangial cell proliferation, whereas AGEs at concentrations of >10 μM markedly inhibit the mesangial cell proliferation^41^. It has also been found that the concentrations of AGEs (AGE-BSA) at 10–50 μg/ml effectively reduced the mouse mesangial cell viability^38^. Yamabe *et al*. found that intracellular AGEs accumulation induced by AGE precursor (500 and 1000 μM glycolaldehyde) caused apoptosis and induced ER stress in chondrocytes^42^. In the present study, we found that 40–160 μg/ml AGEs (AGE-BSA) significantly reduced mesangial cell viability and induced mesangial cell apoptosis. Therefore, the concentrations of AGEs used in this study are reasonable and effectively induce mesangial cell injury.

The present study showed that AGEs induced mesangial cell apoptosis; however, some studies showed that AGEs induced cell proliferation and hypertrophy. Matrix accumulation induced by mesangial cell hypertrophy is already known also an important mechanism in diabetic nephropathy^13^,^43^. It is unclear that why there are two opposite responses in mesangial cells under hyperglycemia condition. Induction of inflammatory response may be one of important reasons that cause AGEs-induced mesangial cells apoptosis^13^. Meek *et al*. found that high level of AGEs induced strong inflammation response through the receptor for AGEs and subsequently induced apoptosis in mesangial cells and podocytes^21^. Furthermore, several studies have shown that ER stress possesses the ability to initiate the reactive oxygen species (ROS) cascades^25^,^44^,^45^. ROS is the most important mechanism for inflammatory response induction in cells^46^. In this study, we found that AGEs markedly induced ER stress and apoptosis in mesangial cells. It is possible that AGEs induce inflammatory response through ER stress-initiated ROS cascades and subsequently increase mesangial cells apoptosis. However, this hypothesis needs to be proved in the future.

Autophagy is a complicated response regulated by cellular nutrient and stress conditions. To adapt environment, autophagy by which plays a protective role or a harmful role depends on different situations. However, the mechanisms in which cells how to decide the role of autophagy were not totally understood. A previous study showed that AGEs induced autophagy through a RAGE/PI3K/AKT/mTOR signaling pathway in cardiomyocytes, which decreased the cell viability in a dose-dependent manner^47^. Autophagy induced by cadmium also impaired the viability of mesangial cells^48^. Atg5 is known to be a gene product required for autophagosome formation. Atg5 cleavage induced by death stimuli has been shown to trigger mitochondria-mediated apoptosis^49^. Leng *et al*. have found that Atg5, but not beclin1, plays a role in ursolic acid-induced autophagic cell death in cervical cancer cells^50^. Nevertheless, autophagy can play the protective roles in osteoblastic cells and podocytes under hyperglycemia circumstance^51^,^52^. Atg5-dependent autophagy has also been found to act the protective effect in paraquat and MPP^+^-induced apoptotic dopaminergic cell death^53^. In the present study, we found that AGEs activated autophagy by ER stress induction in mesangial cells. ER stress inhibition by 4PBA significantly reversed AGEs-induced autophagy, but autophagy inhibition did not influence the AGEs-induced ER stress-related signals. Inhibition of autophagy by Atg5 knockdown could significantly enhance the cytotoxic effect of AGEs on mesangial cells. These results suggest that Atg5-dependent autophagy plays a protective role in AGEs-induced mesangial cell injury.

In conclusion, this study provides evidence to suggest that AGEs induce mesangial cell apoptosis through ER stress induction. Moreover, Atg5-dependent autophagy activated by ER stress plays a protective role in AGEs-induced mesangial cell injury. These findings may offer a new strategy against AGEs toxicity in the kidney. These findings are provocative and deserve further animal study in the future.

## Methods

### Cell culture

Murine glomerular mesangial cell line (SV40 MES13) was purchased from the Bioresource Collection and Research Center (Hsinchu, Taiwan). Cells were cultured in 3:1 mixture of Dulbecco’s modified Eagle’s medium and Ham’s F12 medium contained 5% fetal bovine serum, 14 mM HEPES, 100 U/ml penicillin, and 0.1 mg/ml streptomycin at 37 °C in 5% CO_2_. AGEs (AGE-BSA) were prepared as previously described^4^. The purity of AGEs was determined by liquid chromatography.

### Cell viability assay

Cells were cultured in 24-well plates one day before AGEs treatment. For cell viability evaluation, cells were treated with 0, 10, 20, 40, 80, and 160 μg/ml AGEs for 24 h. BSA (160 μg/ml) treatment was as a negative control for AGEs-induced cytotoxicity. Medium was replaced with PBS contained 0.5 mg/ml 3-(4,5-dimethylthiazol-2-yl)-2,5-diphenyltetrazolium bromide (MTT; Sigma-Aldrich, St. Louis, MO, USA) 24 h after AGEs treatment. The yellow tetrazolium is reduced by mitochondrial succinate dehydrogenase and purple formazan crystal is form in living cells. After 2 h incubation, MTT solution was replaced with dimethyl sulfoxide and then the plates were incubated at room temperature for 30 min with constant agitation to dissolve purple formazan crystal. The absorbance at 570 nm was detected by SpectraMax 190 spectrophotometer (Molecular Devices, Sunnyvale, CA), and the cell viability was calculated.

### Annexin V and propidium iodide (PI) double staining

The numbers of AGEs-induced apoptotic cells were measured by FITC Annexin V Apoptosis Detection Kit (BD Biosciences, San Jose, CA). After cells treated with different concentrations of AGEs for 24 h, cells and media were collected and centrifuged at 1,000 rpm for 5 min at 4 °C. After washed by PBS twice, cells were resuspended in 100 μl 1x binding buffer contained 5 μl PI and 5 μl FITC-annexin V for 15 min. After that, 400 μl 1x binding buffer was replenished. Samples were analyzed by flow cytometry (BD Biosciences). In total 10,000 cells per sample, both early (PI negative and annexin V positive stained cells) and late (PI positive and annexin V positive stained cells) apoptotic cells were analyzed.

### Immunoblotting analysis

After AGEs treatment, cells were washed with PBS twice and then lysed with RIPA buffer contained proteinase inhibitor (1 mM NaF, 1 mM phenylmethanesulfonylfluoride, 1 μg/ml aprotinin and 1 μg/ml leupeptin) on ice for 30 min. The cell lysates were collected and centrifuged at 13,000 rpm for 30 min at 4 °C. The supernatants were collected. The total proteins were denatured by sodium dodecyl sulfate (SDS) sampling buffer and boiled in water for 5 min. SDS polyacrylamide gel electrophoresis (6–15%) was used to separate the total protein samples. The proteins were then transferred from gel to polyvinyl difluoride membrane (Merck Millipore, Billerica, MA, USA). Membranes were blocked by Tris-buffered saline contained 0.1% Tween-20 (TBST) and 5% nonfat milk, and then immersed in sequence of TBST buffer contained primary antibodies for capspase-3, CHOP, phospho-eIF2α, LC3, Atg5, beclin1, and p62 (1:1000; Cell Signaling Technology, Danvers, MA), and secondary horseradish peroxidase antibody (Santa Cruz Biotechnology, San Diego, CA). Finally, signals were detected by Clarity ECL western blotting substrate (BIO-RAD).

### Atg5 siRNA transfection

Small interfering RNA (siRNA) against Atg5 (Stealth Atg5-siRNA; MSS247019, MSS247020, and MSS247021) and negative control siRNA (Stealth RNAi Negative Control kit; 12935110) were purchased from Invitrogen (Carlsbad, CA, USA). Cells were seeded in 6-well plates one day before experiments. The growth medium was replaced with 800 μl OPTI-MEM medium (Invitrogen) before siRNA transfection. OPTI-MEM medium (200 μl) contained siLentFect Lipid (BIO-RAD, Philadelphia, PA) and 80 nM Atg5 siRNA or negative siRNA was added to each well. After 6 h of transfection, OPTI-MEM medium was replaced with 1 ml growth medium.

### Statistics

Data are presented as mean ± SEM for at least three independent experiments. The whole procedure of cellular experiment was repeated at least three separate times with different cell samples. Each repetition was performed in multiple replicates. The significant difference from the respective controls for each experimental test condition was assessed by one-way analysis of variance (ANOVA) followed by post hoc analysis with Bonferroni’s test. A value of *P* < 0.05 was considered statistically significant.

## Additional Information

**How to cite this article**: Chiang, C.-K. *et al*. Involvement of Endoplasmic Reticulum Stress, Autophagy, and Apoptosis in Advanced Glycation End Products-Induced Glomerular Mesangial Cell Injury. *Sci. Rep.* **6**, 34167; doi: 10.1038/srep34167 (2016).

## References

[b1] Yamagishi S., Nakamura K. & Imaizumi T. Advanced glycation end products (AGEs) and diabetic vascular complications. Curr. Diabetes Rev. 1, 93–106 (2005). 18220586 10.2174/1573399052952631

[b2] Lan K. C. . Advanced glycation end-products induce apoptosis in pancreatic islet endothelial cells via NF-κB-activated cyclooxygenase-2/prostaglandin E2 up-regulation. PLoS One 10, e0124418 (2015). 25898207 10.1371/journal.pone.0124418 PMC4405342

[b3] Kim S. Y., Park K. H., Gul R., Jang K. Y. & Kim U. H. Role of kidney ADP-ribosyl cyclase in diabetic nephropathy. Am. J, Physiol. Renal Physiol . 296, F291–F297 (2009). 19073639 10.1152/ajprenal.90381.2008

[b4] Chiu C. Y. . Advanced glycation end-products induce skeletal muscle atrophy and dysfunction in diabetic mice via a RAGE-mediated, AMPK-down-regulated, Akt pathway. J. Pathol. 238, 470–482 (2016). 26586640 10.1002/path.4674

[b5] Angoorani P., Ejtahed H. S., Mirmiran P., Mirzaei S. & Azizi F. Dietary consumption of advanced glycation end products and risk of metabolic syndrome. Int. J. Food Sci. Nutr. 67, 170–176 (2016). 26850840 10.3109/09637486.2015.1137889

[b6] Singh V. P., Bali A., Singh N. & Jaggi A. S. Advanced glycation end products and diabetic complications. Korean J. Physiol. Pharmacol. 18, 1–14 (2014). 24634591 10.4196/kjpp.2014.18.1.1 PMC3951818

[b7] Yu R., Bo H., Villani V., Spencer P. J. & Fu P. The inhibitory effect of rapamycin on toll like receptor 4 and interleukin 17 in the early stage of rat diabetic nephropathy. Kidney Blood Press Res . 41, 55–69 (2016). 26849067 10.1159/000368547

[b8] Yamagishi S. & Matsui T. Advanced glycation end products, oxidative stress and diabetic nephropathy. Oxid. Med. Cell Longev . 3, 101–108 (2010). 20716934 10.4161/oxim.3.2.4 PMC2952094

[b9] Amann K. & Benz K. Structural renal changes in obesity and diabetes. Semin. Nephrol. 33, 23–33 (2013). 23374891 10.1016/j.semnephrol.2012.12.003

[b10] Qin G. . Overexpression of the foxo1 ameliorates mesangial cell dysfunction in male diabetic rats. Mol. Endocrinol. 29, 1080–1091 (2015). 26029993 10.1210/me.2014-1372 PMC5414709

[b11] Hu C. . Insights into the mechanisms involved in the expression and regulation of extracellular matrix proteins in diabetic nephropathy. Curr. Med. Chem. 22, 2858–2870 (2015). 26119175 10.2174/0929867322666150625095407 PMC4863711

[b12] Bhatt K. . Anti-inflammatory role of microrna-146a in the pathogenesis of diabetic nephropathy. J. Am. Soc. Nephrol . (In Press) 10.1681/ASN.2015010111 (2015). PMC4978034 26647423

[b13] Abboud H. E. Mesangial cell biology. Exp. Cell Res. 318, 979–985 (2012). 22414873 10.1016/j.yexcr.2012.02.025

[b14] Steffes M. W., Osterby R., Chavers B. & Mauer S. M. Mesangial expansion as a central mechanism for loss of kidney function in diabetic patients. Diabetes 38, 1077–1081 (1989). 2670639 10.2337/diab.38.9.1077

[b15] Anil Kumar P., Welsh G. I., Saleem M. A. & Menon R. K. Molecular and cellular events mediating glomerular podocyte dysfunction and depletion in diabetes mellitus. Front Endocrinol . 5, 151 (2014). 10.3389/fendo.2014.00151 PMC4174857 25309512

[b16] Khera T., Martin J., Riley S., Steadman R. & Phillips A. O. Glucose enhances mesangial cell apoptosis. Lab. Invest. 86, 566–577 (2006). 16585941 10.1038/labinvest.3700418

[b17] Pesce C. . Glomerular cell replication and cell loss through apoptosis in experimental diabetes mellitus. Nephron 90, 484–488 (2002). 11961409 10.1159/000054738

[b18] Mishra R., Emancipator S. N., Kern T. & Simonson M. S. High glucose evokes an intrinsic proapoptotic signaling pathway in mesangial cells. Kidney Int . 67, 82–93 (2005). 15610231 10.1111/j.1523-1755.2005.00058.x

[b19] Menini S. . Deletion of p66Shc longevity gene protects against experimental diabetic glomerulopathy by preventing diabetes-induced oxidative stress. Diabetes 55, 1642–1650 (2006). 16731826 10.2337/db05-1477

[b20] Yamagishi S. . Advanced glycation end product-induced apoptosis and overexpression of vascular endothelial growth factor and monocyte chemoattractant protein-1 in human-cultured mesangial cells. J. Biol. Chem. 277, 20309–20315 (2002). 11912219 10.1074/jbc.M202634200

[b21] Meek R. L. . Glomerular cell death and inflammation with high-protein diet and diabetes. Nephrol. Dial. Transplant. 28, 1711–1720 (2013). 23314315 10.1093/ndt/gfs579 PMC3707525

[b22] Inagi R. Endoplasmic reticulum stress as a progression factor for kidney injury. Curr. Opin. Pharmacol. 10, 156–165 (2010). 20045381 10.1016/j.coph.2009.11.006

[b23] Dickhout J. G. & Krepinsky J. C. Endoplasmic reticulum stress and renal disease. Antioxid. Redox. Signal. 11, 2341–2352 (2009). 19508129 10.1089/ars.2009.2705

[b24] Oyadomari S. & Mori M. Roles of CHOP/GADD153 in endoplasmic reticulum stress. Cell Death Differ . 11, 381–389 (2004). 14685163 10.1038/sj.cdd.4401373

[b25] Chen B. L. . CCAAT-Enhancer-Binding Protein Homologous Protein Deficiency Attenuates Oxidative Stress and Renal Ischemia-Reperfusion Injury. Antioxid. Redox. Signal. 23, 1233–1245 (2015). 25178318 10.1089/ars.2013.5768

[b26] Xu Y. . Role of the ER stress in prostaglandin E2/E-prostanoid 2 receptor involved TGF-beta1-induced mice mesangial cell injury. Mol. Cell. Biochem. 411, 43–55 (2016). 26463992 10.1007/s11010-015-2567-z

[b27] Kaushal G. P. Autophagy protects proximal tubular cells from injury and apoptosis. Kidney int . 82, 1250–1253 (2012). 23203020 10.1038/ki.2012.337 PMC4068008

[b28] Chen B. L. . Quercetin attenuates renal ischemia/reperfusion injury via an activation of AMP-activated protein kinase-regulated autophagy pathway. J. Nutr. Biochem. 25, 1226–1234 (2014). 25087994 10.1016/j.jnutbio.2014.05.013

[b29] Ko G. J., Bae S. Y., Hong Y. A., Pyo H. J. & Kwon Y. J. Radiocontrast-induced nephropathy is attenuated by autophagy through regulation of apoptosis and inflammation. Hum Exp Toxicol . (In Press) 10.1177/0960327115604198 (2015). 26384705

[b30] Cui J. . Rapamycin protects against gentamicin-induced acute kidney injury via autophagy in mini-pig models. Sci. Rep. 5, 11256 (2015). 26052900 10.1038/srep11256 PMC4459224

[b31] Jakhar R., Paul S., Bhardwaj M. & Kang S. C. Astemizole-Histamine induces Beclin-1-independent autophagy by targeting p53-dependent crosstalk between autophagy and apoptosis. Cancer lett . 372, 89–100 (2016). 26739061 10.1016/j.canlet.2015.12.024

[b32] Chandrika B. B. . Endoplasmic Reticulum Stress-Induced Autophagy Provides Cytoprotection from Chemical Hypoxia and Oxidant Injury and Ameliorates Renal Ischemia-Reperfusion Injury. PLoS One 10, e0140025 (2015). 26444017 10.1371/journal.pone.0140025 PMC4596863

[b33] Liu S. H. . Chemical chaperon 4-phenylbutyrate protects against the endoplasmic reticulum stress-mediated renal fibrosis *in vivo* and *in vitro*. Oncotarget 7, 22116–22127 (2016). 26959118 10.18632/oncotarget.7904 PMC5008348

[b34] Tung W. F. . 4-Phenylbutyric Acid (4-PBA) and Lithium Cooperatively Attenuate Cell Death during Oxygen-Glucose Deprivation (OGD) and Reoxygenation. Cell. Mol. Neurobiol. 35, 849–859 (2015). 25776137 10.1007/s10571-015-0179-5 PMC11486266

[b35] Berg T. J. . The advanced glycation end product Nepsilon-(carboxymethyl)lysine is increased in serum from children and adolescents with type 1 diabetes. Diabetes Care 21, 1997–2002 (1998). 9802757 10.2337/diacare.21.11.1997

[b36] Kilhovd B. K., Berg T. J., Birkeland K. I., Thorsby P. & Hanssen K. F. Serum levels of advanced glycation end products are increased in patients with type 2 diabetes and coronary heart disease. Diabetes Care 22, 1543–1548 (1999). 10480523 10.2337/diacare.22.9.1543

[b37] Zhu W., Li W. & Silverstein R. L. Advanced glycation end products induce a prothrombotic phenotype in mice via interaction with platelet CD36. Blood 119, 6136–6144 (2012). 22431576 10.1182/blood-2011-10-387506 PMC3383021

[b38] Guan S. S., Sheu M. L., Wu C. T., Chiang C. K. & Liu S. H. ATP synthase subunit-β down-regulation aggravates diabetic nephropathy. Sci. Rep. 5, 14561 (2015). 26449648 10.1038/srep14561 PMC4598833

[b39] Yamagishi S. . Advanced glycation end products-induced apoptosis and overexpression of vascular endothelial growth factor in bovine retinal pericytes. Biochem. Biophys. Res. Commun. 290, 973–978 (2002). 11798169 10.1006/bbrc.2001.6312

[b40] Mahali S., Raviprakash N., Raghavendra P. B. & Manna S. K. Advanced glycation end products (AGEs) induce apoptosis via a novel pathway: involvement of Ca^2+^ mediated by interleukin-8 protein. J. Biol. Chem. 286, 34903–34913 (2011). 21862577 10.1074/jbc.M111.279190 PMC3186367

[b41] Geoffroy K., Wiernsperger N., Lagarde M. & El Bawab S. Bimodal effect of advanced glycation end products on mesangial cell proliferation is mediated by neutral ceramidase regulation and endogenous sphingolipids. J. Biol. Chem. 279, 34343–34352 (2004). 15184394 10.1074/jbc.M403273200

[b42] Yamabe S. . Intracellular accumulation of advanced glycation end products induces apoptosis via endoplasmic reticulum stress in chondrocytes. FEBS J . 280, 1617–1629 (2013). 23374428 10.1111/febs.12170

[b43] Chen C. . Polydatin attenuates AGEs-induced upregulation of fibronectin and ICAM-1 in rat glomerular mesangial cells and db/db diabetic mice kidneys by inhibiting the activation of the SphK1-S1P signaling pathway. Mol. Cell. Endocrinol. 427, 45–56 (2016). 26948947 10.1016/j.mce.2016.03.003

[b44] Zeeshan H. M., Lee G. H., Kim H. R. & Chae H. J. Endoplasmic Reticulum Stress and Associated ROS. Int. J. Mol. Sci . 17, 327 (2016). 10.3390/ijms17030327 PMC4813189 26950115

[b45] Song Q., Gou W. L. & Zhang R. FAM3A attenuates ER stress-induced mitochondrial dysfunction and apoptosis via CHOP-Wnt pathway. Neurochem. Int. 94, 82–89 (2016). 26939760 10.1016/j.neuint.2016.02.010

[b46] Rodriguez-Iturbe B., Vaziri N. D., Herrera-Acosta J. & Johnson R. J. Oxidative stress, renal infiltration of immune cells, and salt-sensitive hypertension: all for one and one for all. Am. J. Physiol. Renal Physiol . 286, F606–F616 (2004). 15001451 10.1152/ajprenal.00269.2003

[b47] Hou X. . Advanced glycation endproducts trigger autophagy in cadiomyocyte via RAGE/PI3K/AKT/mTOR pathway. Cardiovasc. Diabetol. 13, 78 (2014). 24725502 10.1186/1475-2840-13-78 PMC3998738

[b48] Wang S. H., Shih Y. L., Ko W. C., Wei Y. H. & Shih C. M. Cadmium-induced autophagy and apoptosis are mediated by a calcium signaling pathway. Cell. Mol. Life Sci. 65, 3640–3652 (2008). 18850067 10.1007/s00018-008-8383-9 PMC11131605

[b49] Luo S. & Rubinsztein D. C. Atg5 and Bcl-2 provide novel insights into the interplay between apoptosis and autophagy. Cell Death Differ . 14, 1247–1250 (2007). 17431417 10.1038/sj.cdd.4402149

[b50] Lengm S. . Ursolic acid promotes cancer cell death by inducing Atg5-dependent autophagy. Int. J. Cancer 133, 2781–2790 (2013). 23737395 10.1002/ijc.28301

[b51] Yamahara K. . The role of autophagy in the pathogenesis of diabetic nephropathy. J. Diabetes Res. 2013, 193757 (2013). 24455746 10.1155/2013/193757 PMC3877624

[b52] Bartolome A. . Autophagy impairment aggravates the inhibitory effects of high glucose on osteoblast viability and function. Biochem. J. 455, 329–337 (2013). 23981124 10.1042/BJ20130562

[b53] Garcia-Garcia A. . Impairment of Atg5-dependent autophagic flux promotes paraquat- and MPP^+^-induced apoptosis but not rotenone or 6-hydroxydopamine toxicity. Toxicol. Sci. 136, 166–182 (2013). 23997112 10.1093/toxsci/kft188 PMC3829573

